# Adaptive Responses in Severe Acute Malnutrition: Endocrinology, Metabolomics, Mortality, and Growth

**DOI:** 10.3390/nu17172864

**Published:** 2025-09-04

**Authors:** Laura Page, Elizabeth McCain, Michael Freemark

**Affiliations:** 1 Division of Pediatric Endocrinology, Duke University Medical Center, 3000 Erwin Road, Durham, NC 27005, USA; laura.page@duke.edu (L.P.); elizabeth.mccain@duke.edu (E.M.); 2 Duke Molecular Physiology Institute, Duke University Medical Center, Durham, NC 27705, USA

**Keywords:** growth hormone, cortisol, insulin, insulin-like growth factor, leptin, adiponectin, GLP-1, peptide YY, cytokines, lipolysis, fatty liver, protein kinetics, stunting, wasting, mid-upper arm circumference, microbiome, environmental enteropathy

## Abstract

Malnutrition afflicts millions of the world’s children and predisposes to death from diarrhea and infectious diseases. Children with severe acute malnutrition (SAM) are at highest risk. Our review of the endocrinology and metabolomics of SAM implicates critical roles for white adipose tissue and its regulatory hormones and growth factors in the adaptation to nutritional deprivation and the restoration of metabolic homeostasis: white adipose provides substrates and energy for hepatic glucose production and cardiopulmonary and central nervous system function, and products of fat metabolism inhibit muscle glucose uptake and utilization and spare muscle protein. Collectively, these effects maintain glucose availability for the brain, red blood cells, and renal medulla and conserve muscle mass. White adipose tissue also secretes leptin, which facilitates the immune response and may protect against mortality from infection. Euglycemia and survival in SAM are thereby prioritized over linear growth, which is suppressed owing to inhibition of insulin-like growth factor 1 production and action. Diversion of energy from growth serves to maintain essential bodily functions in critically ill malnourished children, who have limited energy reserves. Thus, short-term reductions in growth rate have adaptive benefits in SAM. Under favorable conditions, clinical and metabolic recovery are accompanied by catch-up growth, which can mitigate, and in many cases reverse, the stunting of growth in childhood. Nevertheless, clinical recovery can be complicated by preferential accrual of central fat and a relative deficiency of lean/skeletal mass, with potential long-term complications including insulin resistance, glucose intolerance, and metabolic syndrome.

## 1. Introduction

**Figure 1:**
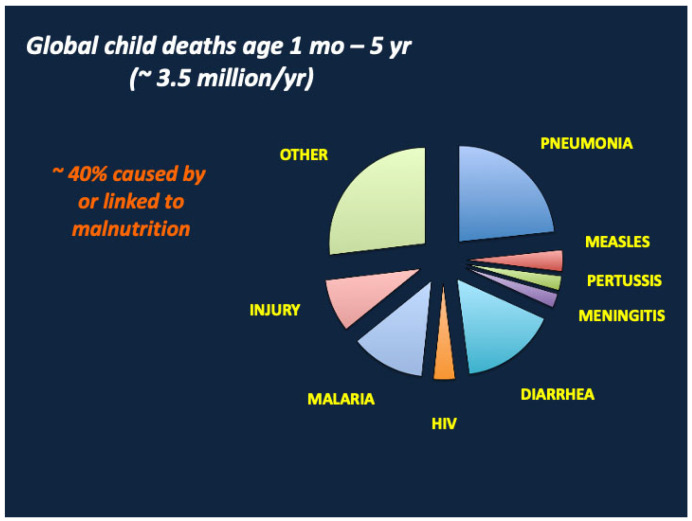
Malnutrition increases the risks of death from diarrhea, pneumonia, sepsis, and critical viral and parasitic diseases including measles, malaria, and HIV. Adapted from Liu et al. [3].

**Figure 2:**
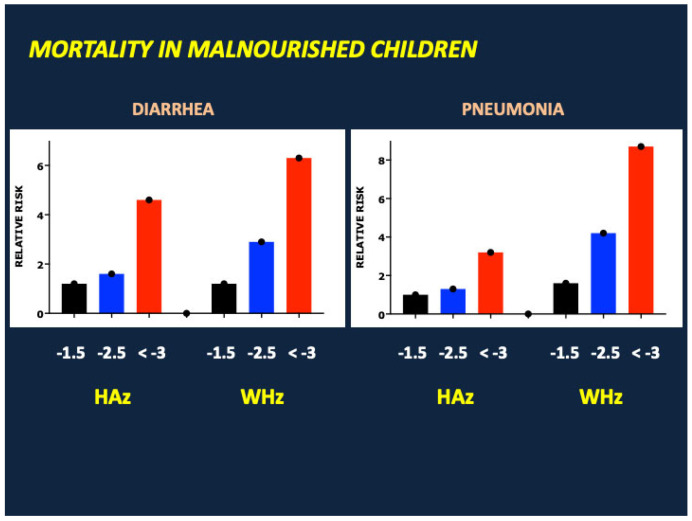
The risks of dying from diarrhea, pneumonia, and various other infectious diseases increase in proportion to the deficits in height for age z (HAz) and weight for height z (WHz). Adapted from Walker et al. [4].

Malnutrition is an ancient scourge [1] most prevalent, and most devastating, in under-resourced communities confronting drought, crop failure, disease, or war. Despite widespread advances in agriculture and food production, sanitation, industrial development, transportation, communication, and medical care during the past 250 years, many of the world’s children remain at high risk of malnutrition and its acute and long-term complications including growth failure, cognitive impairment, vaccine resistance, immune deficiency, and death from diarrhea and infectious diseases [2]. Global estimates suggest that malnutrition causes or contributes to 40% of the 3.5 million deaths each year in infants and young children aged 1 month to 5 years [3] (Figure 1). As shown in Figure 2, mortality rates correlate with the severity of malnutrition, as defined by reductions in height and weight for length standard deviation (z) scores, and are highest in severe acute malnutrition (SAM), a life-threatening condition associated with severe weight loss [weight for height z (WHz) < −3 and/or mid-upper arm circumference (MUAC) < 11.5 cm] and/or nutritional edema [4]. SAM is estimated to afflict 13.6 to 19 million children under the age of five years, with highest prevalence in South Asia (India, Pakistan and Bangladesh); Southeast Asia (Indonesia, Sri Lanka, East Timor and Cambodia); Oceania (Papua New Guinea and the Solomon Islands); Sub-Saharan Africa; and various war zones including Syria, Sudan, Afghanistan, Yemen, and Gaza [5,6]. Factors predisposing to SAM include poverty, drought, famine, food insecurity, overcrowding, lack of adequate sanitation, inadequate vaccination, and low birth weight, the latter often linked to maternal malnutrition, suboptimal pregnancy weight gain, and/or maternal smoking [7,8].

Many previous studies have characterized the pathologic complications of SAM, which include micronutrient deficiencies, steatosis, hypoglycemia, growth failure/stunting, and immune deficiency as well as death from diarrhea and infectious diseases. In this narrative (non-systematic) review, we focus on the adaptive responses to nutrient deprivation that maintain euglycemia and cardiopulmonary and central nervous system function and increase the likelihood for survival in critically ill patients. Adaptive response is here defined as a modification of a bodily function or physical or behavioral trait that enhances performance or improves survival in an individual or species subjected to environmental stress.

Our primary objectives were to review the literature regarding: (a) the endocrinology and metabolomics of SAM; (b) the pathogenesis of growth failure/stunting in children with SAM; and (c) clinical and biochemical markers associated with mortality in SAM. We used the information gleaned from our analysis to address critically three important questions in the assessment of children with SAM:

- How do the endocrine and metabolic responses to nutrient deprivation promote survival and recovery from SAM?
- Why might growth suppression (“stunting”) represent an evolutionary adaptation/tradeoff that facilitates recovery from SAM?
- Can we identify biomarkers that predict mortality in SAM? What are their roles in the adaptation to SAM and the defense against life-threatening infectious diseases? And how are the circulating levels of critical biomarkers regulated by changes in body composition prior to and during treatment of SAM?

## 2. Methods

We undertook a review of the literature (in English) published between January 1965 and July 2025. Publications were identified using a search of the PubMed and Cochrane Library databases as well as general online search engines. Given the broad scope of the discussion, our review largely focused on human studies. Nevertheless, groundbreaking investigations in experimental animals were also analyzed. All publications were evaluated critically for scientific rigor, impact, and relevance to our subject.

Keywords employed for the literature search included the terms malnutrition, marasmus, kwashiorkor, wasting, stunting, growth failure, enteropathy, fasting, starvation, inflammation, and HIV, alone or in combination with others words or phrases including low birth weight, small for gestational age, metabolomics, amino acids, protein kinetics, fat metabolism, fatty liver disease, microbiome, micronutrients, and the various hormones, growth factors, and cytokines discussed in the manuscript. Our review included studies that characterize: (1) factors that predispose to SAM; (2) the endocrinology and metabolomics of malnutrition; (3) the metabolic differences between malnourished children with marasmus (non-edematous) and kwashiorkor (edematous); (4) the pathogenesis of nutritional enteropathy and inflammation in SAM and potential roles of the microbiome; (5) the pathogenesis of growth failure/stunting in malnutrition; (6) and clinical and biochemical markers linked to inpatient or outpatient mortality in SAM. Exclusion criteria included duplicate reports, studies focusing solely on adults or adolescents, or those without nutrition-related outcomes.

The literature on SAM published during the past 60 years is extensive. Likewise, the range and number of papers describing the biochemical and hormonal control of metabolism and growth is vast. Thus, our review is non-systematic. But we have endeavored to analyze and assess all recent papers on SAM and those historical papers considered most impactful and relevant to our discussion. We reference critical investigations and reviews of the control of metabolism and growth where relevant. It should be noted that our postulates regarding the roles of adipose tissue in the adaptation to malnutrition were formulated *after* our review of the literature on SAM. Thus, our selection of papers for review and discussion was not governed by preexistent hypotheses or biases.

## 3. Clinical Phenotypes of Severe Acute Malnutrition

SAM in children manifests as distinct but overlapping clinical phenotypes. Marasmus is characterized by severe wasting, with loss of fat and muscle mass. Growth failure/stunting is common. The characteristic feature of Kwashiorkor is edema; associated findings include skin desquamation, hair hypopigmentation, and hepatomegaly with steatosis. Wasting and growth failure are variable, and muscle mass may be maintained to at least some degree. These findings suggest that the pathogeneses of marasmus and kwashiorkor differ in certain critical respects. Nevertheless, both marasmus and kwashiorkor can be accompanied by a small bowel enteropathy, with dysbiosis (alterations in the gastrointestinal microbiome), inflammation, villous atrophy, increases in gastrointestinal permeability, macro- and micro-nutrient deficiencies, and immune deficiency [9,10], and some children have an “overlap” syndrome characterized by severe wasting with edema (“marasmic kwashiorkor”).

Children with SAM often present with anorexia, likely a consequence of gastrointestinal and systemic release of inflammatory cytokines that inhibit food intake, such as interleukin 1 beta, tumor necrosis factor alpha, and interleukin 6 [11,12]. High levels of gastrointestinal hormones (glucagon-like peptide 1 and peptide YY) at presentation [13] may act in concert with inflammatory cytokines to suppress appetite (see below). The transient anorexia of malnutrition and acute infection may have certain adaptive benefits, including: (a) a limit on the energy expenditure required for food intake, digestion, absorption, and metabolism; (b) a decrease in the intake of iron, which promotes bacterial replication; and (c) induction of autophagy, with lipolysis, fatty acid oxidation, and ketogenesis providing substrates and energy critical for survival (see [11,12,14,15,16] and discussion below).

## 4. The Endocrinology and Metabolomics of Severe Acute Malnutrition

Pioneering studies of children with SAM were conducted during the 1970s, 1980s, 1990s, and early 2000s by leading investigators including Waterlow, Whitehead, Golden, James, Alleyne, Young and Picou, Pimstone and Becker, and Manary, Badaloo, and Jahoor. Collectively, their investigations showed that SAM is accompanied by striking changes in amino acid metabolism, protein kinetics, immune function, and hepatic fat deposition, as well as deficiencies in macro- and micro-nutrients and antioxidants such as glutathione. Endocrinologic responses were shown to include fasting hypoinsulinemia, hypercortisolemia (particularly in patients with concurrent infection), and high levels of growth hormone [17,18,19,20,21,22,23,24,25,26,27,28].

Here we highlight recent seminal investigations that provide more comprehensive and integrated analyses of the endocrine and metabolic adaptations to SAM. Bartz et al. studied 75 malnourished infants and toddlers in Kampala, Uganda [13]. They ranged from 5 months to 5 years of age; 57% were edematous, 24% were HIV+, and one in 10 had malaria. Blood was obtained at the time of hospital admission, 2 weeks after nutrient supplementation with F75 and F100 formulas, and 6–10 weeks after discharge on Ready-To-Use Therapeutic Food (RUTF).

**Figure 3:**
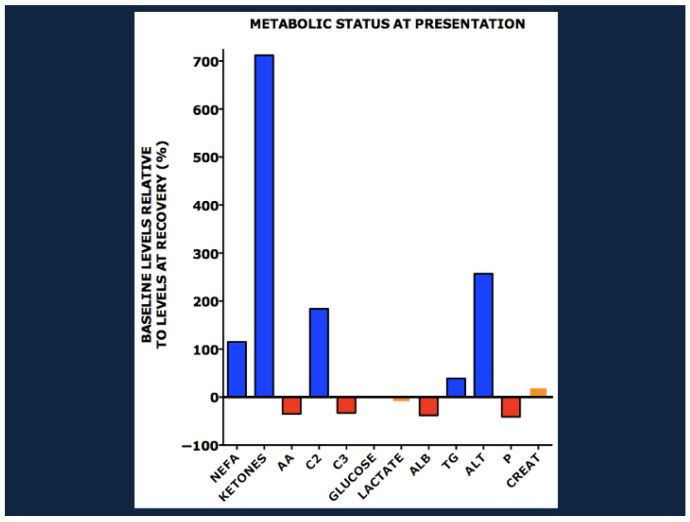
Metabolite levels in children with severe acute malnutrition (SAM) at baseline relative to those at outpatient recovery. NEFA, non-esterified fatty acids; AA, total amino acids; C2, acetylcarnitine; C3, propionylcarnitine; Alb, albumin; TG, triglycerides; ALT, alanine aminotransferase; P, phosphorus; creat, creatinine. From Bartz et al. [13], with copyright permission from the publisher.

Figure 3 shows levels of metabolites at baseline relative to those at outpatient recovery.

At baseline, patients had high levels of non-esterified fatty acids (NEFA), ketones, beta hydroxybutyrate, and even-chain acylcarnitines including acetylcarnitine (C2), indicating active lipolysis and fatty acid oxidation. In contrast, the levels of albumin, amino acids (including alanine, methionine, glutamic acid/glutamine, lysine, serine, proline, threonine, glycine, tyrosine, tryptophan, ornithine, citrulline, arginine, and the branch chain amino acids), and propionylcarnitine (C3), a by-product of branched chain amino acid catabolism, were low, reflecting decreased amino acid availability, inhibition of proteolysis (particularly in edematous patients, see below) and reductions in amino acid catabolism. Triglycerides and ALT were high, implicating hepatic fat deposition. Phosphorus was low, but lactate, creatinine, and baseline blood sugar were normal.

**Figure 4:**
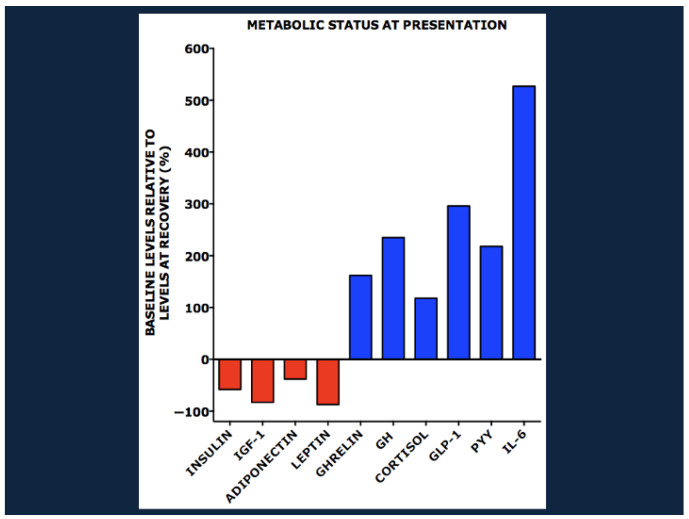
Hormone, growth factor, and cytokine levels in children with SAM at baseline relative to those at outpatient recovery. IGF-1, insulin-like growth factor 1; GH, growth hormone; GLP-1, glucagon-like peptide 1; PYY, peptide YY; IL-6, interleukin 6. From Bartz et al. [13] with copyright permission from the publisher.

These metabolic derangements were accompanied by striking changes in the plasma concentrations of various hormones, growth factors, and inflammatory cytokines (Figure 4). At baseline there were low levels of insulin, IGF-1, and two adipocytokines: adiponectin, a determinant of insulin sensitivity, and leptin, a marker of white adipose tissue reserve. In contrast there were striking increases in the levels of ghrelin, growth hormone, and cortisol as well as the gastrointestinal hormones glucagon-like peptide 1 (GLP-1) and peptide YY (PYY), the cytokine interleukin 6 (IL-6), and the inflammatory marker C-reactive peptide.

**Figure 5:**
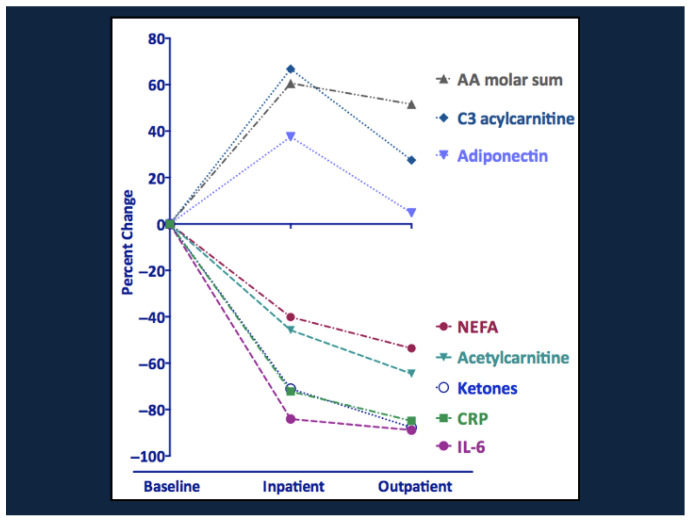
Changes in metabolite and cytokine levels in SAM during clinical recovery. AA, amino acids; C3, propionylcarnitine; NEFA, non-esterified fatty acids, CRP, C-reactive protein; IL-6, interleukin 6. From Bartz et al. [13], with copyright permission from the publisher.

**Figure 6:**
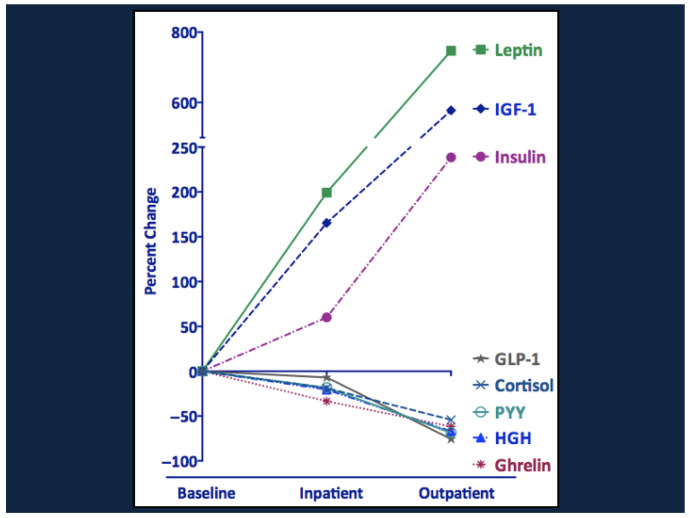
Changes in hormones and growth factors in SAM during clinical recovery. IGF-1, insulin-like growth factor 1; GLP-1, glucagon-like peptide 1; PYY, peptide YY; HGH, growth hormone. From Bartz et al. [13], with copyright permission from the publisher.

Serial changes in the various metabolites, hormones, growth factors, and cytokines during the course of therapy and recovery are shown in Figure 5 and Figure 6. Note the striking decline in NEFA, ketones, acetylcarnitine, CRP, and IL-6 and the rise in amino acids and propionylcarnitine (C3). The increase in C3 suggests that the rise in branch chain amino acids during recovery reflects their increased availability from the diet rather than a block in amino acid catabolism. There were also transient increases in total and high molecular weight adiponectin, which increases insulin sensitivity and may promote branch chain amino acid catabolism [29]. Metabolic recovery was accompanied by striking increases in insulin, leptin, and IGF-1 and a fall in the levels of ghrelin, GH, cortisol, GLP-1, and peptide YY.

Marked reductions in amino acids and an increase in even-chain acylcarnitines at presentation were also noted in malnourished Nigerian children ages 6–48 months, one-half of whom had edema [30]. Additional findings included low levels of phospholipids in the phosphatidylcholine and phosphatidylethanolamine families as well as several oxylipins, derived from oxidation of long chain polyunsaturated fatty acids.

Likewise, a study of 343 Bangladeshi infants and young children (age 6–36 months) with SAM found high levels of NEFA, ketones, and even-chain acylcarnitines with normal glucose and lactate levels at presentation [31]. There were low levels of various amino acids including alanine, glutamine/glutamic acid, tyrosine, arginine, ornithine, citrulline, and the branch chain amino acids, and low levels of branch chain ketoacids and propionyl carnitine. Consistent with the findings of Bartz et al. [13] and other investigations [32] there were low levels of insulin, leptin, and IGF-1, with high levels of CRP, IL-6, and IGF binding protein 2, which can inhibit IGF-1 signaling [33,34,35,36], as well as various inflammatory proteins including CRP, IL-6, IL-1β, TNFα, CD40L, and the ubiquitin conjugating enzyme E2 N (UBE2N). Clinical recovery was accompanied by reductions in NEFA, ketones, and even chain acylcarnitines and increases in amino acids, branch chain ketoacids, and propionylcarnitine. Insulin, IGF-1, and leptin rose.

Collectively, these studies suggest that untreated severe acute malnutrition is accompanied by high rates of lipolysis, fatty acid oxidation, and ketogenesis, with nitrogen preservation associated with reductions in amino acid catabolism. Fasting euglycemia is maintained in most cases despite severe nutritional deprivation, though a minority of malnourished children, often critically ill with superimposed infection(s), present with fasting hypoglycemia or impaired glucose tolerance [37,38]. The metabolic adaptations are associated with, and orchestrated by, striking changes in the production and actions of various hormones, growth factors, and inflammatory cytokines.

## 5. Comparative Metabolomics in Marasmus and Kwashiorkor

As noted previously, SAM in children manifests as distinct but overlapping clinical phenotypes, with varying degrees of wasting and edema. Classic features of edematous patients include skin desquamation, hair hypopigmentation, and hepatomegaly with steatosis. While some children have an overlap syndrome with severe wasting and edema, the phenotypic differences between marasmus and kwashiorkor suggest differences in pathogenesis. A number of investigators have therefore compared the metabolic status of patients with marasmus and kwashiorkor at presentation and their metabolic responses to treatment.

### 5.1. Protein and Lipid Metabolism

Manary et al. studied whole-body protein kinetics in 21 malnourished children in Malawi; half had HIV [22]. Rates of proteolysis and protein synthesis were higher in marasmus than in kwashiorkor. A subsequent investigation found that rates of protein turnover were lower in children with marasmus with or without infection than in infected, well-nourished controls [39]. Likewise, Jahoor and Badaloo et al. found lower rates of proteolysis and lower levels of branch chain amino acids, methionine, phenylalanine, and tyrosine in edematous than in non-edematous malnourished children [40]. Rates of protein synthesis in kwashiorkor were comparable to those in marasmus at baseline. However, nutritional recovery was accompanied by higher rates of protein synthesis in kwashiorkor. Badaloo and Jahoor also found lower rates of fatty acid oxidation in kwashiorkor than in marasmus, reflecting in part a lower level of carnitine [41].

A more comprehensive analysis of metabolic changes in marasmus and kwashiorkor was presented by DiGiovanni et al. [42]. As predicted by Badaloo and Jahoor [41], the levels of C14:1, C18:1 and the ratio of acetylcarnitine (C2) to free carnitine were higher in marasmus than in kwashiorkor, suggesting higher rates of beta oxidation of fatty acids. Likewise, as in Jahoor et al. [40], amino acids and albumin were lower in kwashiorkor than in marasmus but rose in both groups following treatment. Certain lysophosphatidylcholines were low at baseline and increased following clinical stabilization. In contrast, sphingomyelins were low at presentation but did not recover over the course of the study.

In sum, these findings suggest that fatty acid oxidation and protein turnover are lower in edematous patients with kwashiorkor than in wasted patients with non-edematous marasmus. Nevertheless, rates of protein turnover and amino acid catabolism are lower in marasmus as well as kwashiorkor in comparison with well-nourished children.

### 5.2. One Carbon Metabolism and the Pathogenesis of Edema

It should be noted that the pathogenesis of edema in kwashiorkor is poorly understood. There is no clear-cut correlation between total protein intake or plasma albumin concentrations and the onset, severity, or resolution of edema; nevertheless, the diet of children with kwashiorkor is characteristically low in protein, and severe hypoalbuminemia is more common in kwashiorkor than in marasmus. May et al. [43] note that the clinical phenotype and metabolic abnormalities of kwashiorkor mirror those of experimental animals subjected to diets deficient in nutrients critical for one carbon metabolism, including B vitamins, folate, choline, and methionine, and they find lower levels of methionine, homocysteine, cysteine, and glutathione in edematous than in non-edematous children. Refining an idea first proposed by Golden [44,45], they hypothesize that a deficiency of methionine may reduce the synthesis of sulfated glycosaminoglycans critical for endothelial function, promoting the leakage of plasma proteins like albumin from the intravascular space into interstitial fluids. A defect in one-carbon metabolism is also linked to hypomethylation of DNA and variable gene expression in edematous malnutrition [46].

### 5.3. Hepatomegaly, Steatosis, and Hepatic Dysfunction

As noted above, hepatomegaly, macrovesicular steatosis, and hepatic dysfunction are reported to be far more common in kwashiorkor than in marasmus [47,48]. However, a recent investigation showed marked steatosis in non-edematous children with severe wasting, 3 of 4 of whom were infected with HIV [49]. In the absence of chronic infection or hepatotoxins like aflatoxin, the steatosis and hepatic dysfunction in kwashiorkor and marasmus usually resolve with adequate supplementation of protein and micronutrients.

Factors contributing to steatosis and hepatic dysfunction in SAM include: (a) an excess of substrate (free fatty acids) derived from exaggerated lipolysis; (b) a partial deficit in beta oxidation, resulting from deficiencies of protein, micronutrients, and ATP in marasmus and, in kwashiorkor, a reduction in the number of hepatic mitochondria and peroxisomes and low levels of carnitine [49,50,51,52]. Other potential factors include an increase in the ratio of unconjugated to conjugated bile acids, which may cause *cholestatic* liver injury and increase the levels of ALT [53,54]. Reductions in the synthesis of Apolipoprotein B 100 and assembly of VLDL, which are required for hepatic export of triglycerides, have been hypothesized to cause hepatic steatosis in malnourished children; however, VLDL synthesis at baseline in children with edematous and non-edematous SAM was comparable to that measured following clinical recovery [55].

## 6. The Effect of Concurrent HIV Infection

Concurrent infection with HIV increases markedly the risk of death in severe acute malnutrition [13,56]. In a secondary analysis of malnourished Ugandan infants and children, the baseline triglycerides, even-chain acylcarnitines, ketones, valine, phenylalanine, interleukin 2, and TNFα were higher in HIV-infected children than in HIV-negative children, while plasma alanine, leptin, and adiponectin were lower [57]. Leptin levels rose in all patients following nutritional intervention, but adiponectin levels remained depressed in HIV-infected children.

Adiponectin levels were also markedly reduced in patients with HIV infection in a secondary analysis of malnourished children with Kenya and Malawi [58]. Consistent with the findings of Mody et al. [57], there was upregulation of pathways involving VLDL assembly and hepatic triglyceride export [58]. Additional correlates of HIV infection included heightened expression of various inflammatory pathways and down-regulation of the adipokine ZAG (zinc-alpha-2-glycoprotein), which is implicated in the dyslipidemia and glucose intolerance of the metabolic syndrome in obese adults.

## 7. Enteropathy and the Role of the Microbiome

SAM in infants and young children is associated with developmental immaturity and reduced diversity of the gastrointestinal microbiome [59]. It is currently unclear if this “dysbiosis” results from maternal malnutrition and/or dysbiosis, childhood feeding practices, lack of sanitation, superimposed infection, antibiotic exposure, and/or gut mucosal barrier dysfunction, and its precise relationship to small bowel enteropathy and the complications of SAM remains poorly understood.

Several lines of evidence suggest that dysbiosis may cause gastrointestinal inflammation, malabsorption of macro- and micro-nutrients, disruption of the mucinous barrier, and systemic exposure to gastrointestinal pathogens, with downstream effects on amino acid metabolism, weight gain, and linear growth [9,10,60]. For example, gnotobiotic mice transplanted with the stool microbiome of children with kwashiorkor had exaggerated weight loss on a low protein diet (but not on standard mouse chow) and lower fecal levels of methionine and cysteine than those transplanted with stool from their healthy twins [61]. Moreover, transplantation of fecal microbiota of malnourished infants into germ-free mice reduced rates of weight gain and accrual of lean mass during the subsequent 4–5 weeks [62]. Finally, a complementary diet designed to promote colonization with “healthy” microbiota increased serum insulin, IGF-1, and branch-chain amino acids in germ-free mice and short term (4 weeks) weight gain (+0.3 WHz) in young Bangladeshi children (age 12–18 months) with moderate acute malnutrition [31]. A subsequent 3-month study showed higher rates of weight gain (+0.13 WHz) in malnourished children receiving a microbiota-directed complementary food than in those receiving a standard ready-to-use supplemental food; there were no effects on height for age z or MUAC [63].

On the other hand, microbiome maturity in 259 malnourished children in Malawi correlated only weakly with weight for height Z score (WHz, r = 0.14–0.17); the association with height for age z (r = 0.10–0.12) was even less robust [62]. Small bowel biopsies of malnourished hospitalized Zambian children with enteropathy and persistent diarrhea showed no correlations between duodenal villous height, villous perimeter, or plasma Glucagon-like Peptide 2 and weight for age z (WAz), weight for length z (WHz), length for age z (LAz), or mid-upper arm circumference (MUAC) [64]. Duodenal biopsies of stunted (LAz < −2) Bangladeshi children (mean age 18 months) and age-matched children “at risk for stunting” (LAz < −1–2) found nonspecific duodenitis in more than 90% and no difference in rates of villous blunting or atrophy between the groups [65]. Moreover, in a combined cohort of 352 Pakistani, Bangladeshi, and Zambian infants and young children with varying degrees of stunting and wasting and American children with normal duodenal histopathology, there were no correlations between HAz, WAz, or WHz and a composite histologic score comprising villus blunting, intraepithelial lymphocytes, goblet and Paneth cell depletion, and intramucosal Brunner’s glands [66].

Experimental evidence suggests that dysbiosis in some children is a consequence, rather than a cause, of the metabolic derangements in SAM. For example, a low protein diet in mice alters the composition of the microbiome, promotes GI inflammation, reduces villous height, and limits branch chain amino acid catabolism [67]. Moreover, diets deficient in one-carbon nutrients such as methionine or choline can promote intestinal inflammation, increase mucosal permeability, and raise plasma levels of inflammatory markers like TNFα in experimental animals [43].

## 8. Synthesis: How Do the Endocrine and Metabolic Responses to Nutrient Deprivation Promote Survival and Recovery from SAM?

**Figure 7:**
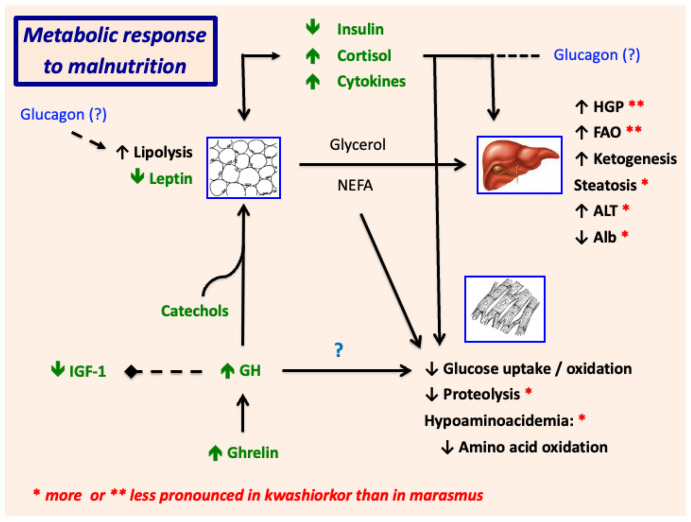
Hormonal and metabolic responses to SAM. GH, growth hormone; IGF-1, insulin-like growth factor 1; NEFA, non-esterified fatty acids; HGP, hepatic glucose production; FAO, fatty acid oxidation; ALT, alanine aminotransferase; Alb, albumin. Certain metabolic responses are * more or ** less pronounced in kwashiorkor than in marasmus.

**Figure 8:**
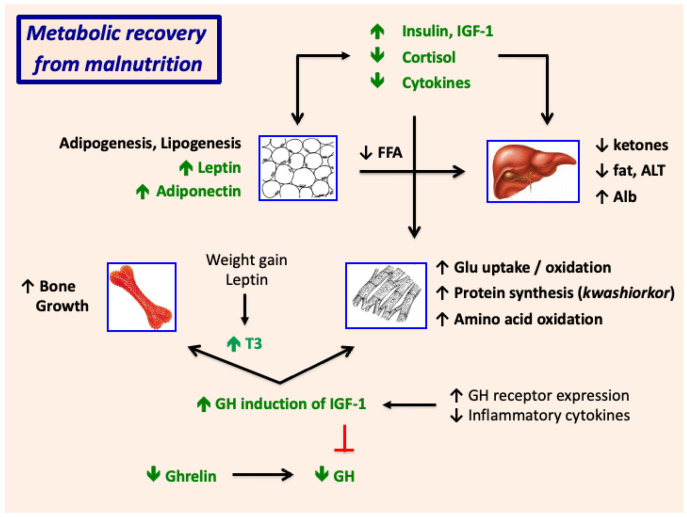
Metabolic recovery from SAM. GH, growth hormone, IGF-1, insulin-like growth factor 1; T3, tri-iodothyronine; FFA, free (non-esterified) fatty acids; ALT, alanine aminotransferase; Alb, albumin; Glu, glucose.

Figure 7 and Figure 8 depict schematically the roles of hormones, growth factors, and cytokines in the pathogenesis of SAM and restoration of metabolic homeostasis during clinical recovery.

### 8.1. Hormonal and Metabolic Responses to Nutrient Deprivation

Nutrient deprivation and the release of inflammatory cytokines trigger a catabolic, autophagic response comprising lipolysis, fatty acid oxidation, and ketogenesis, which provide substrates and energy/ATP for hepatic glucose production and cardiopulmonary function. Together with reductions in skeletal muscle glucose uptake and oxidation, these adaptations also maintain euglycemia (in most patients) for supply of the brain [68], red blood cells, liver, and renal medulla. Thus, *the catabolic response is essential for survival in SAM*. Nevertheless, lipolysis-driven increases in FFA, in combination with decreases in peroxisomal beta oxidation, can promote hepatic steatosis and a transient increase in liver enzymes.

Rates of muscle proteolysis and protein turnover are decreased, particularly in edematous children, to limit or prevent further loss of muscle protein and muscle mass. In combination with reductions in amino acid availability from low dietary protein intake and/or GI protein loss, the attenuation of proteolysis reduces amino acid oxidation and utilization. In critically ill children with SAM, the depletion of glucogenic amino acids such as alanine, glutamate, isoleucine, and valine may precipitate a fall in blood sugar. Concurrent infection(s) may also predispose to fasting hypoglycemia; conversely, the associated cytokine surge may in some cases attenuate insulin secretion and limit glucose tolerance [37].

The metabolic adaptations of SAM are orchestrated by changes in the expression and action of various hormones, growth factors, and adipocytokines. Lipolysis is driven by a ghrelin-induced rise in growth hormone (GH) and is facilitated by increases in catecholamines [69,70] and cortisol and, most importantly, a fall in insulin. The lipolytic effects of GH are mediated by induction of hormone-sensitive lipase, inactivation of PPARgamma, and down-regulation of FSP27 (fat-specific protein 27) [71,72]. Through inhibition of lipoprotein lipase, GH also reduces adipocyte uptake of free fatty acids [73].

In response to fasting or severe illness, high levels of cortisol can acutely induce expression of adipose triglyceride lipase and hormone sensitive lipase and promote lipolysis in subcutaneous (but not visceral) fat. The lipolytic effects of cortisol may be largely permissive, exerted in opposition to insulin and in synergy with catecholamines [74,75]. The rise in plasma cortisol in SAM is stress-related but may also be triggered in part by a fall in leptin: hypoleptinemia in experimental animals increases pituitary ACTH secretion and thereby raises plasma corticosterone concentrations [76,77,78]. In humans, however, the role of leptin in the regulation of the HPA axis is less clear [79].

The roles of glucagon and the gastrointestinal hormones in the pathogenesis of SAM are also poorly understood. While glucagon promotes lipolysis and hepatic glucose production in humans and experimental animals, the levels of glucagon in one study *rose* during clinical recovery from SAM; in a second study, glucagon levels in kwashiorkor at diagnosis were comparable to those in healthy controls but far lower than those in marasmus (which were highly variable) [80,81]. Likewise, the high levels of GLP-1 and peptide YY at baseline [13] seem in some ways counterintuitive, as these hormones stimulate insulin secretion, inhibit glucagon release, increase insulin sensitivity, and suppress appetite [82,83,84,85]. It is possible that the enteropathy, dysbiosis, and inflammation of SAM cause a maladaptive rise in the gastrointestinal hormones; alternatively, their appetite-suppressive effects, exerted in concert with inflammatory cytokines, and their ability to slow gastric emptying, might have adaptive benefits, facilitating the catabolic response to illness (see Clinical Phenotypes of SAM, above).

Given the low levels of glucogenic amino acids and normal levels of lactate at baseline, the primary substrate for gluconeogenesis in children with SAM at presentation appears to be glycerol; the energy for gluconeogenesis is derived from oxidation of free fatty acids. In addition to providing glycerol and free fatty acids through induction of lipolysis, GH inhibits insulin-dependent glucose uptake and oxidation in skeletal muscle. Potential mechanisms explaining these effects include: a reduction in muscle insulin receptor expression; serine (as opposed to tyrosine) phosphorylation of insulin receptor substrate 1; and downregulation of PI3 kinase activity owing to induction of its p85alpha regulatory subunit [86]. Studies of experimental mice with a targeted deletion of the skeletal muscle GH receptor [87] suggest that the counterregulatory effect of GH may also be mediated through induction of SOCS2, which inhibits insulin signaling.

On the other hand, studies in human adults demonstrate that GH action in skeletal muscle can be blocked by treatment with Acipimox, an inhibitor of lipolysis [88,89]. The resulting block in free fatty acid generation can restore insulin-dependent glucose uptake because free fatty acids compete with glucose for utilization by skeletal muscle cells and down-regulate pyruvate dehydrogenase activity [90,91]. Moreover, re-esterification of free fatty acids in muscle generates diacylglycerol and ceramides, both of which inhibit insulin signaling and the translocation of Glut4 to the cell surface [92]. These observations suggest that fatty acids mediate the effects of GH on insulin-dependent glucose uptake and utilization in skeletal muscle.

Muscle proteolysis is suppressed in kwashiorkor (and to a lesser extent in marasmus) despite low levels of insulin and IGF-1 and high levels of cortisol. We do not know if the high levels of GH limit proteolysis and thereby prevent further muscle protein loss in untreated children with SAM. Several studies conducted under basal conditions suggest that GH stimulates skeletal muscle protein synthesis but has little or no effect on muscle proteolysis [93,94,95]. Nevertheless, skeletal muscle proteolysis in human adults is exaggerated during fasting if GH is suppressed, and branch-chain amino acid levels decline following GH replacement [90]. It remains unclear if this represents a direct or an indirect effect on skeletal muscle; however, the nitrogen retaining effect of GH and its inhibition of muscle proteolysis are also blocked by Acipimox, implicating a critical role for free fatty acids. Indeed, a number of studies suggest that energy provided by free fatty acids and ketones spares muscle protein during periods of prolonged fasting [96,97,98,99]. In sum, the high levels of GH and the catabolic responses in adipose tissue and liver maintain euglycemia and limit muscle loss in children with SAM.

The growth promoting effects of GH are mediated through systemic (primarily hepatic) production of IGF-1 and differentiation and expansion of IGF-responsive growth plate chondrocytes [100,101]. GH may also exert direct, IGF-independent effects on chondrocyte growth [102]. Despite marked elevations of GH at presentation, the circulating levels of IGF-1 in SAM are suppressed. Together with a fall in GH receptor expression (as reflected in a decrease in the level of the GH binding protein) [63], this finding implicates a resistance to GH production of IGF-1. The resistance may be selective for the growth promoting effects of GH, as its effects on lipid metabolism are IGF-1-independent. Selective growth hormone resistance therefore plays a critical adaptive role in which energy production and survival are prioritized over linear growth. Mechanisms explaining the resistance to IGF-1 generation are discussed in more detail in the Section 9 on growth below.

### 8.2. Metabolic Recovery from Malnutrition

Nutritional recovery is accompanied by an increase in insulin and reductions in cortisol, ghrelin, GH, and inflammatory cytokines (Figure 6 and Figure 8); in concert, these promote lipogenesis and reduce lipolysis and increase white adipose tissue mass. Increases in fat mass explain the progressive increases in plasma leptin concentrations over time. The fall in GH following nutrient repletion together with induction of white adipogenesis, may explain the transient increase in adiponectin, which promotes hepatic fatty acid oxidation and increases insulin sensitivity [103,104,105,106,107]. In combination, the striking changes in hormone production and action during recovery limit hepatic ketogenesis, reduce hepatic fat stores, and increase insulin-dependent glucose uptake and utilization in skeletal muscle.

Clinical recovery is accompanied by gradual reversal of the GH resistance to IGF-1 production. The rise in IGF-1, in concert with the rise in insulin and fall in cortisol, stimulates protein synthesis and limits proteolysis and thereby promotes accrual of muscle mass [108]. IGF induction of chondrocyte proliferation stimulates an increase in long-bone growth. IGF-1-dependent bone growth is potentiated by a rise in tri-iodothyronine (T3), which stimulates clonal expansion of chondrocyte progenitor cells and promotes the differentiation of hypertrophic chondrocytes [109,110]. The increase in plasma T3 may be driven in part by the increase in leptin, which activates the hypothalamic-pituitary-thyroid axis and modulates activity of the deiodinases in a tissue-dependent manner [111,112,113]. The rise in leptin during clinical recovery may also contribute to the concurrent rise in plasma IGF-1 [114,115].

## 9. Growth Failure and Stunting in SAM

An extensive body of literature shows that nutritional deficits imposed during fetal and early postnatal (<24 months) life can cause linear growth failure (low growth velocity) and “stunting”, defined as height <−2 SD below the mean for sex and age. Critical determinants of stunting in infancy include prematurity, low birth length, low birth weight, poverty, lack of parental education, and male sex; in combination, prematurity and intrauterine growth restriction (IUGR) increase the risk of postnatal stunting by 2–7 fold [9,116,117,118,119]. This may explain in part the high rates of stunting in developing countries, where mean length for age standard deviation (z) score at birth approximates −0.5, and low birth weight (LBW) is six times more common than in the developed world [120]. Maternal health plays a central role: factors predisposing to low birth weight and length and early childhood stunting include a history of pre-pregnancy malnutrition and maternal short stature; early age at pregnancy; sub-optimal pregnancy weight gain; and placental insufficiency [9,116,117,118,121,122].

A longitudinal analysis of weight gain and linear growth in infants and young children in rural Gambia demonstrates that stunting is commonly preceded by episodes of nutritional wasting [123]. Likewise, childhood growth failure/stunting in postnatal life is a common complication of medical illness or nutritional deficiencies. Many authors thus consider childhood stunting a state of chronic nutritional deprivation accompanied by severe long-term co-morbidities including cognitive deficits, school failure, loss of human productivity, and an increased risk of mortality before 24 months of age [119,124].

Because growth failure and stunting can reflect the interaction of environmental, social, and cultural determinants, medical illness, genetic determinants, and maternal and childhood dietary deficiencies, the prevalence of stunting has practical utility as a metric of population health and quality of life. While useful on a population scale, the anthropometric definition of stunting, and its purported relation to chronic malnutrition and disability, are problematic when applied to individuals. First, approximately 2.5% of normal, healthy children and adults in any given population will be considered “stunted” using a standard definition of height for age <−2 SD. Second, the correlation between low height for age and disability in malnourished children is continuous, without a distinct break-point at −2 SD [125]. Third, as discussed by Scheffler et al., many stunted children have no clinical or anthropometric evidence of malnutrition at the time of assessment [126]. Finally, short stature per se, as in children with idiopathic short stature, familial short stature, and constitutional delay of growth and puberty, is not accompanied by deficits in cognitive, behavioral, or social function and does not increase the risks of mortality in childhood or adolescence. Likewise, early childhood stunting alone, in the absence of wasting, is not associated with a higher risk of mortality in the first two years of life [119].

The specific mechanisms by which malnutrition causes growth failure and postnatal stunting are poorly understood, though inadequate or inconsistent energy and protein intake, micronutrient deficiencies, recurrent infection(s), and local and systemic inflammation likely play critical roles. Deficits in protein are accompanied by pre- and postnatal growth failure in humans and experimental animals), while deficiencies of micronutrients such as iron and zinc can suppress appetite and reduce small intestinal villous surface area and gastrointestinal absorptive capacity [127,128,129,130,131]. Inadequate intake and malabsorption of zinc in children with SAM may impede IGF-1 production and action and may thereby reduce the rate of linear growth [67,128,132]. Nevertheless, effects of zinc supplementation on linear growth in children *without* SAM are inconsistent and limited in magnitude [133,134,135].

Inflammation of the small intestine in children with enteropathy and systemic infection are accompanied by high levels of C-reactive protein (CRP) and release of inflammatory cytokines that impair production and action of chondrocyte growth factors. For example, malnourished Ugandan and Bangladeshi children had high levels of interleukin 6 (IL-6), which blocks growth hormone (GH) induction of insulin-like growth factor 1 (IGF-1) and inhibits IGF action at the growth plate [13,31,136,137]. Likewise, IL-6 levels in Zimbabwean infants (birth-18mo) correlated inversely with IGF-1 and were elevated soon after delivery in a subset of subjects with low birth weight [138].

A central role for suppression of IGF-1 production and action in the growth failure of SAM is supported by a study of enteric enteropathy in Bangladeshi children (age 12–18 months) with moderate acute malnutrition [63]. Correlates of stunting included low levels of IGF-1 as well as the acid labile subunit (which binds IGF-1), soluble Klotho (which increases sensitivity to IGF-1), and leptin, which can stimulate an increase in circulating IGF-1 in leptin-deficient children [114,115]. Conversely, stunting was associated with high levels of IGF binding protein 2 (which can inhibit IGF signaling in growth plate chondrocytes), PAPPA (which cleaves IGF binding proteins), and a number of inflammatory cytokines including CXCL11.

**Figure 9:**
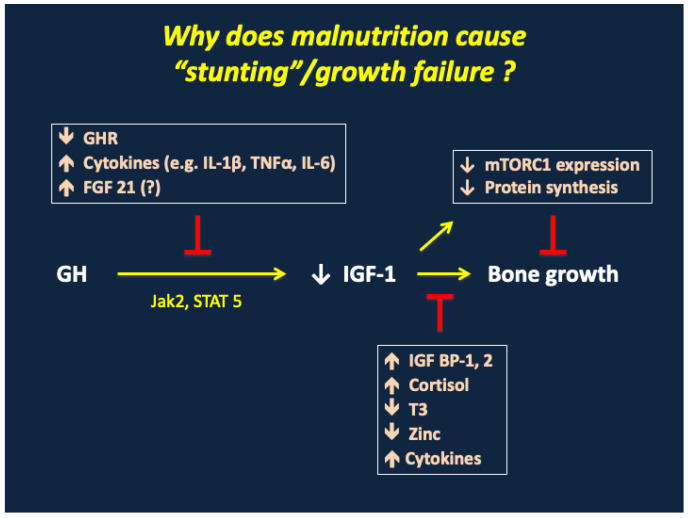
Growth failure/stunting in SAM. GHR, growth hormone receptor; IL-1β, interleukin-1 beta; TNFα, tumor necrosis factor alpha, IL-6, interleukin 6; FGF21, fibroblast growth factor 21; GH, growth hormone; IGF-1, insulin-like growth factor 1; mTORC1, mammalian target of rapamycin complex 1; IGF BP-1, 2, insulin-like growth factor binding proteins 1 and 2; T3, tri-iodothyronine.

As noted previously, the levels of IGF-1 in SAM at presentation are suppressed despite marked elevations of GH. This resistance to GH-dependent IGF-1 production results in part from a reduction in GH receptor expression: stunting in children with enteropathy was associated with low levels of the GH binding protein, the circulating extracellular domain of the GH receptor [63]. But as shown in Figure 9, IGF-1 generation is also blocked by defects in the phosphorylation of Jak2 and STAT5, critical intracellular mediators of GH signaling. IGF-dependent chondrocyte proliferation, which requires nutrient-dependent mTORC1 expression and protein synthesis, is in turn blocked by high levels of IGF binding proteins 1 and 2, cortisol, and inflammatory cytokines [139,140,141]. Growth suppression is exacerbated by low levels of T3 and, in some cases, by a deficiency of zinc [67,109,110,128,132,134]. The role of Fibroblast Growth Factor 21 (FGF21), which can inhibit IGF-1 production in malnourished animals, is currently unclear, as baseline levels in malnourished and stunted children are extraordinarily variable and correlate only weakly with anthropometric data [142,143,144,145].

### 9.1. Why Might Growth Suppression (“Stunting”) Represent an Evolutionary Adaptation/Tradeoff That Facilitates Recovery from SAM?

In untreated children with SAM, the transient resistance to GH-dependent IGF-1 generation coincides with an acute catabolic response comprising lipolysis, fatty acid oxidation, and ketogenesis. These metabolic adaptations, which are evolutionarily conserved in mammals [71,73,142], provide substrates and energy for hepatic glucose production and cardiopulmonary and central nervous system function and prioritize euglycemia and survival over linear growth. Energy required for growth must be diverted to maintain essential bodily functions in critically ill malnourished children, who have limited energy reserves. Thus, a *reduction in growth rate has adaptive benefits in SAM* and would be expected to abate or resolve following nutritional recovery, resolution of medical illness, and restoration of metabolic homeostasis. And this indeed appears to be the case, as revealed by examination of long-term growth trajectories in malnourished children.

**Figure 10:**
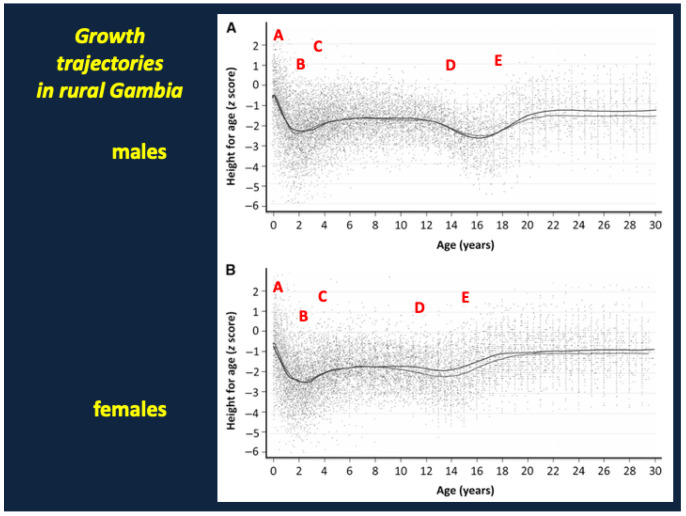
Growth trajectories in rural Gambia. From Prentice et al. [146], with copyright permission from the publisher. See the text for explanation of the annotations (A–E), which have been added by the authors of this manuscript.

Figure 10 shows anthropometric data (36,828 measurements) from children, adolescents and young adults in rural Gambia obtained by the Medical Research Council’s field station during the past 60 years [146]. The original figure has been annotated to identify salient features. Note (A) that mean height for age z score (HAz) is low at birth (~0.75 SD). There is a precipitous fall in mean HAz (B) to a nadir approximating −2.4 z at age 24–30 months of age. This period of growth failure is followed by *accelerated* catch-up growth (C), with HAz stabilizing in the range of −1.5 z through age 12 years in boys and 11 years in girls. Thereafter one notes what appears to be an exaggerated pre-pubertal decline (D) in growth velocity, a pattern not unusual in otherwise healthy children with constitutional delay. This is followed by a delayed and prolonged, but largely adequate, pubertal growth acceleration (E). Mean final height, which approximates −0.9 to −1.2 SD, is not achieved in the boys until age 21 years and in the girls until age 18–19 years. The mean heights of the men and women in this study no longer fall in the stunted range.

Likewise, the mean heights of adult (age 27–32 years) survivors of childhood marasmus and kwashiorkor in Jamaica were at or near the normal range (males −0.60 z, females +0.01 z); their mean HAz scores in infancy had approximated −4.0 z (males) and −3.4 z, respectively [147]. Similar findings have been reported in a survey of growth data from 5 countries in the COHORTS study: children who were most stunted at 24 months of age had the greatest gain in HAz through adulthood [148]. And in rural Senegal, the adult HAz scores of formerly stunted women (mean −0.9 z) and men (mean −1.4 z) were considerably greater than their HAz scores prior to age 5 years (mean −2.8 z for both) [149].

Collectively, these data demonstrate that early growth failure can be mitigated, and in many cases reversed, by robust catch-up growth and a delayed and prolonged adolescent growth phase.

### 9.2. Failure of Catch-Up Growth and Long-Term Metabolic Complications

Failure of catch-up growth and persistent stunting in children with a history of pre- or early postnatal growth failure might be explained by: (a) inadequate reserve of, or epigenetic changes in, cells critical for growth including myocytes and chondrocytes; (b) long-term defects in small intestinal maturation and growth; and/or (c) recurrent bouts of nutrient deprivation, infection, and cytokine excess associated with small bowel and systemic inflammation [9]. When nutritional recovery is delayed or incomplete or there is recurrent infection, the child is likely to have persistent wasting and secondary growth failure and will remain at higher risk for re-admission and death [150] (see also Section 10 on Mortality below). 

Following recovery from infection and nutrient repletion there is a tendency for weight to be deposited or conserved as fat mass in preference to lean body mass. Differential tissue accretion is highly variable and depends in part on growth and weight gain in utero, nutritional status at admission, dietary intake, and the rate of catch-up weight gain [68,151,152]. Older adolescent and adult male survivors of childhood SAM are more likely than females to show preferential storage of fat in the abdominal/visceral regions and long-term deficits in height (relative to community controls) [153].

The imbalance between fat mass and lean body/skeletal mass likely contributes to metabolic dysfunction with age. Waist-hip ratio (WHR, 0.94) and hemoglobin A1c (HbA1c, 4.6%) were slightly higher in adult survivors of SAM in the Democratic Republic of Congo than in unexposed community controls (WHR 0.91, HbA1c 4.2%) [154]. Highest rates of abdominal obesity were observed in those with mixed marasmic-kwashiorkor, though the mild increase in HbA1c was noted in all survivors of SAM [155]. Among adult (age 17–50 yr) survivors of SAM in Jamaica, glucose intolerance (mild) was more common in those with a history of marasmus (with severe generalized wasting) than in those with a history of kwashiorkor (in which muscle mass may be better preserved) [147]. Limited evidence suggests that the glucose intolerance results in part from a reduction in post-prandial insulin secretion [156].

As yet, no studies have demonstrated higher risks of overt diabetes or cardiovascular disease in survivors of SAM. Future investigations of diabetes following SAM will need to clearly differentiate the effects of malnutrition in infancy and childhood from those of combined pre- and postnatal growth restriction; low birth weight and intrauterine growth restriction are major determinants of type 2 diabetes in adolescents and adults [157,158,159,160].

## 10. Biomarkers That Predict Mortality in SAM: White Adipose Tissue Energy Reserves and the Role of Leptin

Children with SAM are often critically ill; hospitalization is required for complications including anorexia, severe edema, metabolic dysfunction, infection, persistent vomiting, or seizures. An analysis of 19 studies, most performed in sub-Saharan Africa, found rates of inpatient mortality ranging from 3.7 to 41.4% (mean 15.7%); mortality following discharge ranges from 0.6 to 11.7% [161,162].

Clinical factors predisposing to mortality in SAM include prematurity and intrauterine growth restriction, age less than 24 months, severe wasting (with very low WHZ and MUAC), HIV infection, persistent diarrhea, sepsis, and shock [13,57,163,164]. Hypoglycemia and impaired glucose tolerance can develop in critically ill hospitalized children and may contribute to mortality in some children with SAM [37,38].

A number of investigations have noted correlations between circulating biomarkers and mortality in SAM. A study of 79 hospitalized Malawian children found associations between inpatient mortality and high levels of inflammatory cytokines including GCSF, IL-1ra, IL-6, IL-2, TNFα, TNFβ, and IL-13; these are presumably derived from small bowel and systemic inflammation and infection [165]. Likewise, a study of post-discharge mortality in malnourished Kenyan children found strong associations with various inflammatory proteins including CRP, LBP, TNFα, IL-8, IL-15, IP10, and GCSF [166].

**Figure 11:**
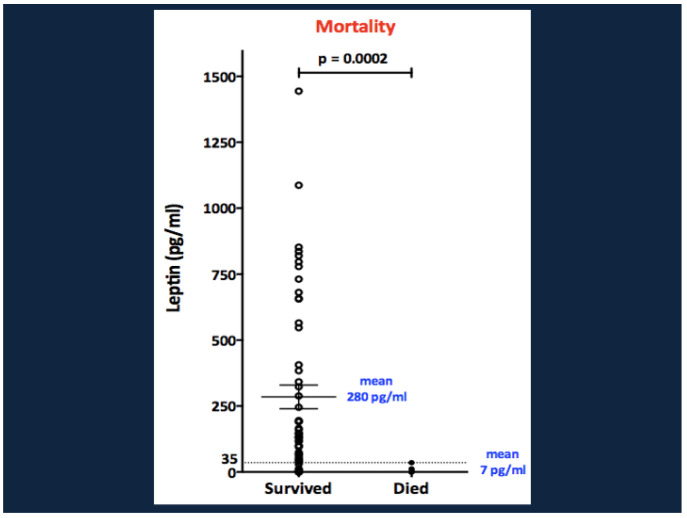
Leptin levels at baseline and inpatient mortality in children with SAM. From Bartz et al. [13], with copyright permission from the publisher.

Univariate analysis of biomarkers in Ugandan malnourished infants found positive correlations between inpatient mortality and baseline levels of IL-2, IL-6, TNFα, and peptide YY. Conversely, mortality correlated negatively with levels of adiponectin [13,57]. But the major biochemical correlate of mortality, in edematous as well as non-edematous children, was a low level of leptin. All 9 children with baseline leptin <35 pg/mL died. In contrast, 45/54 (84%) of children with baseline leptin >35 survived (Figure 11). In multivariate logistic regression analysis, the combination of HIV and baseline weight for height z score and plasma leptin level explained 67% of mortality risk. Severe hypoleptinemia (<35 pg/mL) at diagnosis also strongly predicted early post-discharge mortality in a cohort of 1778 Kenyan infants and young children with SAM [166].

### 10.1. Why Might Hypoleptinemia Associate with, or Predispose to, Mortality in SAM?

Leptin is synthesized primarily by white adipose tissue, and its circulating level in non-fasted subjects serves as a marker of white adipose tissue mass. Accordingly, the plasma concentrations of leptin in malnourished children correlate far more strongly with weight for height z score than with height for age z or MUAC [167,168]. It is likely that hypoleptinemic children have diminished white adipose tissue stores at the time of presentation; we speculate that they can generate and oxidize free fatty acids acutely but deplete their adipose reserves under continuing stress. Depletion of white adipose stores is postulated to limit the ability to *sustain* energy production in children with concurrent infections or other severe medical illnesses; this would be expected to increase the child’s risk of death. Proving this thesis will require additional investigation; nevertheless, a critical role for fat mass in the defense against mortality in suggested by previous studies of Bantu children with SAM and well-nourished Ugandan and Kenyan children with severe pneumonia [169,170].

Hypoleptinemia resulting from depletion of white adipose may also increase the risk of death from infection through loss of immune competence, as leptin promotes T cell development, proliferation, and function and regulates many aspects of innate and acquired immunity. Consequently, leptin deficiency may impede the immune response to bacterial, viral, and parasite pathogens.

### 10.2. Role of Leptin in Innate Immunity

Leptin activates NK cells, neutrophils, and monocytes/macrophages while enhancing chemotaxis for neutrophils, basophils, and eosinophils [171,172,173]. In macrophages, leptin promotes phagocytosis and oxidative burst [171,172,174]. Leptin also drives pro-inflammatory cytokine production by monocyte/macrophages and dendritic cells [171,172,175]. Exposing human monocytes to short-term leptin in vitro increased production of TNFα, IL-6, and IL-1β [176]. Similarly, short-term leptin exposure stimulated IFN-γ release from human NK cells [173]. Leptin promotes dendritic cell maturation and enhances the ability of dendritic cells to induce CD4^+^ T-cell proliferation [172,177]. Importantly, leptin promotes survival of NK cells, neutrophils, basophils, eosinophils, dendritic cells through reduced apoptosis [171,172,178,179,180,181].

### 10.3. Role of Leptin in Adaptive Immunity

Leptin is critical to T-cell maturation and differentiation in the thymus. Leptin promotes thymopoiesis and naïve CD4^+^ T-cell proliferation [172,182,183]. Thymic atrophy occurs in malnutrition, a state of leptin-deficiency, and can be reversed with nutrient supplementation [184,185,186]. High levels of thymocyte apoptosis as well as thymic atrophy are similarly noted in leptin deficient mice and are improved by exogenous leptin administration [187,188].

Leptin exerts direct effects on T cells by acting as a pro-inflammatory cytokine to drive T-cell polarization to Th1 cells, which produce IFN-γ and IL-2 [172,175]. Similarly, leptin induces secretion of pro-inflammatory cytokines, including IL-6, IL-10, and TNFα, from B cells [189]. Leptin also decreases rates of B-cell and mature T-cell apoptosis [172,175].

Consistent with the role of leptin in adaptive immunity, children with leptin deficiency due to a leptin gene mutation showed reduced numbers of CD4^+^ T cells along with increased CD8^+^ and B cells, resulting in a low CD4^+^/CD8^+^ ratio [112]. Treatment with recombinant human leptin, reversed these findings, increasing CD4^+^ T-cell levels, decreasing CD8^+^ and CD19 B cells, and normalizing the CD4^+^/CD8^+^ T cell ratio [112]. Similarly, lymphocyte proliferative responses and cytokine production, particularly Th1 or pro-inflammatory cytokine production such as IFN-γ, improved with recombinant human leptin therapy [112].

### 10.4. Genetic and Acquired Defects in Leptin Production or Action Increase the Risks of Morbidity & Mortality from Infectious Disease

Consistent with the role of leptin in immune function, children with leptin deficiency or leptin receptor deficiency, due to genetic mutations in leptin or the leptin receptor, respectively, have increased risks of severe pulmonary and gastrointestinal infections and mortality from infectious diseases [190]. In one family, children with presumed leptin deficiency, based on pedigree, had 25.4 times increased odds of death compared to their non-affected family members [191]. These deaths, which occurred during childhood, were due to infections [191]. In a cohort of children with severe monogenic obesity, mortality rates were 26% and 9% for leptin or leptin receptor deficiency, respectively, while no deaths were reported in comparably obese children with melanocortin 4 receptor (MC4R) deficiency [190]. In the same cohort, approximately 40% of children with leptin or leptin receptor deficiency developed gastrointestinal infections with severe diarrhea requiring hospitalization and 38–55% had recurrent respiratory infections including pneumonia [190].

Generalized lipodystrophy, a disorder characterized by adipopenia and severe hypoleptinemia, is also associated with increased risks of mortality from pneumonia and other severe infectious diseases [192,193,194]. An international, retrospective study evaluating the natural history of non-HIV-related lipodystrophy, found a mean time to death of 51.2 years in patients with generalized lipodystrophy [195]. Of the 10 patients with generalized lipodystrophy who died during the observation period, three were attributed to infections, including pneumonia, and/or sepsis [195]. A separate study from Brazil analyzed causes of death in twenty patients with congenital generalized lipodystrophy [194]. Mean age at death was 27.1 years with 35% of deaths caused by infections, most commonly pneumonia [194]. A systematic review of pediatric patients with non-HIV-related lipodystrophy found a 6.5% mortality rate among patients with congenital generalized lipodystrophy at a mean age of 12.5 years [193]. Most deaths were due to respiratory infections [193]. A Spanish cohort found a 16.6% mortality rate among patients with generalized lipodystrophy, with half of deaths caused by respiratory infections and a mean time to death of 55.3 years [192]. However, respiratory infections did not appear to contribute to mortality in a cohort of Turkish patients with generalized lipodystrophy [196]. Instead, most deaths were related to end-stage renal disease (ESRD) and sepsis [196].

Collectively, these findings suggest that white adipose tissue reserves and leptin play critical roles in the defense against life-threatening infections. As discussed previously, children with SAM are prone to death from HIV, pneumonia, and other infectious diseases. The increased risks of mortality in SAM are likely related, at least in part, to an inability to sustain energy production during the course of infection as well as an impaired immune response to bacterial and viral pathogens.

## 11. Limitations

The limited number of longitudinal cohorts and metabolomic datasets may constrain the generalizability of the findings. Our schematic models will not apply to all children with SAM and our postulates require additional study for confirmation (or refutation). For example, biochemical responses in malnourished children with kwashiorkor and “marasmic kwashiorkor” and their long-term metabolic complications differ from those in children with marasmus. The origin and pathogenesis of these phenotypic and biochemical differences remain poorly understood, and variations in hormonal responses have not been fully explored. Likewise, the roles of adipose tissue reserve and leptin in the defense against infection may differ among patients with marasmus, who have severe generalized wasting, and kwashiorkor, who are more likely to have steatosis and preservation of muscle mass. In that vein, the protective roles of white adipose reserves and leptin in survival of children with SAM have not yet been demonstrated *directly*; additional study is clearly warranted. Similarly, a direct role for growth hormone in the conservation of muscle mass has not yet been established. Moreover, our suggestion that GLP-1 and PYY contribute to transient anorexia as an adaptive response to SAM requires validation. Finally, there are likely numerous other hormones, growth factors, and cytokines that contribute to the adaptive response to nutrient deprivation in infants and children. Thus, our analysis, while broadly supported by the available data, is at times oversimplified and in places exploratory and incomplete.

## 12. Conclusions, Gaps in Knowledge, and Future Investigations

Children with SAM are prone to micronutrient deficiencies, steatosis, hypoglycemia, growth failure, immune deficiency, and death from diarrhea and infectious diseases. Here we have highlighted the adaptive responses to nutrient deprivation that maintain euglycemia and cardiopulmonary and central nervous system function and increase the likelihood for survival in critically ill patients. Our review implicates a central role for white adipose tissue metabolism and the secretion of leptin in the adaptation to, and recovery from, severe nutrient deprivation. The hormonal and metabolic responses to SAM prioritize euglycemia, survival, and central nervous system function over energy-demanding linear growth, which is suppressed by inhibition of IGF-1 production and action. Thus, short-term growth failure in children with SAM has adaptive benefits. Growth failure/stunting can be mitigated, and in many cases reversed, if nutritional support is restored and maintained and the subsequent medical course remains otherwise uncomplicated. When the clinical course is complicated by persistent or recurrent bouts of nutrient deprivation and/or infection, the child is likely to have persistent wasting and secondary growth failure and will remain at higher risk for re-admission and death. Clinical recovery can be complicated by preferential accrual of central fat and a relative deficiency of lean/skeletal mass, with potential long-term complications including insulin resistance, glucose intolerance, and the metabolic syndrome.

Despite extensive analysis there remain critical gaps in our understanding of the pathogenesis of SAM and its attendant short- and long-term complications. Important questions include:

1. Why are prematurity, intrauterine growth restriction, and low birth weight associated with higher risks of mortality in SAM? Do they serve as markers or proxies of longstanding (and future) poverty, food insecurity, limited access to medical care, and/or lack of sanitation or education in the family and community, or might they exert epigenetic effects) that increase the susceptibility to nutrient deprivation or infection?
2. Small bowel enteropathy and dysbiosis have been implicated with the pathogenesis of SAM and its complications including sepsis and growth failure. What are the roles of micronutrient and protein deficiencies in the development of enteropathy and dysbiosis in SAM?
3. What explains the high levels of GLP-1 and PYY in children with SAM? Do the appetite-suppressive effects of these gastrointestinal hormones have adaptive benefits in the response to nutrient deprivation? Does GLP-1 modulate the local immune responses to enteropathy?
4. The roles of other gastrointestinal hormones in the adaptation to SAM are unclear. For example, Glucagon-like peptide 2 (GLP-2) regulates epithelial growth, permeability, and nutrient absorption in the gastrointestinal tract. Available studies find high circulating levels of GLP-2 in infants and young children with acute diarrhea but not in those with persistent diarrhea or SAM [197]; yet, GLP-2 levels are low in stunted infants [197,198] and, in one study, correlated negatively with mortality in SAM [199]. Thus, additional investigation of its effects on weight gain, linear growth, and survival in SAM are warranted.
5. What are the roles of gastrointestinal vs. systemic inflammation in the pathogenesis of growth failure in children with SAM?
6. FGF21 is thought to play a central role in the adaptation to fasting in experimental animals, while Growth Differentiation Factor 15 (GDF15) may inhibit food intake in chronic illness and cancer [200,201,202,203]. What are the roles of FGF21, GDF15, and other novel hormones and growth factors in the control of appetite, weight gain, and linear growth in SAM?
7. What factors determine the rates of catch-up growth and weight gain in malnourished children with growth failure/stunting? Can final height in stunted children be predicted by parental target height independent of parental nutritional status?
8. As noted previously, some studies find that adult survivors of SAM are predisposed to mild glucose intolerance, dyslipidemia, and metabolic syndrome, but increases in rates of overt diabetes or cardiovascular disease have not yet been demonstrated. Future studies should clarify the roles of central adiposity, sarcopenia, and pancreatic beta cell dysfunction in the long-term metabolic complications of SAM. It will be critical to distinguish complications of malnutrition in infancy and childhood from those of combined pre- and postnatal growth restriction.
9. Leptin has important immunomodulatory roles, and a deficiency of leptin or its receptor predisposes to morbidity and mortality from severe infection. Future studies should determine if the hypoleptinemia of SAM modifies the local immune response to small bowel dysbiosis as well as the systemic response to infection.
10. Finally, we considered the theoretical benefits and risks of leptin therapy in malnourished children. In children and adults with lipodystrophy, which is associated with severe hypoleptinemia, recombinant leptin has metabolic benefits including reductions in hepatic fat content, fasting glucose, HbA1c, ALT, AST, and triglyceride levels [204,205,206]. However, treatment causes weight loss and reductions in body fat mass and lean body mass owing to a decline in food intake [207]. Reductions in weight, fat mass, and lean body mass would be maladaptive in children with SAM. Moreover, leptin levels rise spontaneously and dramatically during clinical recovery from SAM. Thus, the potential risks of leptin therapy in SAM appear to outweigh its potential benefits.

## Data Availability

No new data generated.

## Abbreviations

The following abbreviations are used in this manuscript:

AA	Total Amino Acids
Alb	Albumin
ALT	Alanine aminotransferase
ATP	Adenosine Triphosphate
C2	Acetylcarnitine
C3	Propionylcarnitine
Creat	Creatinine
CRP	C-Reactive Protein
FAO	Fatty Acid Oxidation
FFA	Free fatty acid
FGF21	Fibroblast growth factor 21
FSP27	Fat-specific protein 27
GCSF	Granulocyte colony stimulating factor
GDF15	Growth Differentiation Factor 15
GH	Growth hormone
GLP-1	Glucagon-like peptide 1
GLP-2	Glucagon-like peptide 2
HAz	Height for Age z-score
HGP	Hepatic Glucose Production
IFN-γ	Interferon Gamma
IGF-1	Insulin-like growth factor 1
IL-1β	Interleukin-1 beta
IL-13	Interleukin-13
IL-15,	Interleukin-15
IL-1ra	Interleukin-1 receptor antagonist
IL-2	Interleukin-2
IL-6	Interleukin-6
IL-8	Interleukin-8
IP10	Also known as CXCL10
Jak2	Janus kinase 2
LAz	Length for age z-score
LBP	Lipopolysaccharide-binding protein
mTORC1	Mammalian Target of Rapamycin Complex 1
MUAC	Mid-upper arm circumference
NEFA	Non-esterified fatty acids
NK cells	Natural Killer cells
P	Phosphorus
PAPPA	Pregnancy-associated plasma protein-A
PPAR gamma	Peroxisome proliferator-activated receptor gamma
PYY	Peptide YY
RUTF	Ready-to-Use Therapeutic Food
SAM	Severe Acute Malnutrition
SD	Standard deviation
SOCS2	Suppressor of cytokine signaling 2
STAT5	Signal Transducer and Activator of Transcription 5
TG	Triglycerides
T3	Tri-iodothyronine
TNFα	Tumor Necrosis Factor alpha
UBEN2N	Ubiquitin conjugating enzyme E2 N
VLDL	Very low density lipoprotein
WAz	Weight for age z-score
WHz	Weight for Height z-score
ZAG	zinc-alpha-2-glycoprotein
